# Influence of Classroom-Level Factors on Implementation Fidelity During Scale-up of Evidence-Based Interventions

**DOI:** 10.1007/s11121-022-01375-3

**Published:** 2022-04-29

**Authors:** Katie Massey Combs, Pamela R. Buckley, Marion Amanda Lain, Karen M. Drewelow, Grace Urano, Suzanne E. U. Kerns

**Affiliations:** 1 Institute of Behavioral Science, University of Colorado Boulder, 1440 15th St, Boulder, CO 80309 USA; 2 Department of Anthropology, University of California San Diego, La Jolla, CA 92092 USA; 3 Graduate School of Social Work, University of Denver, Denver, CO 80208 USA; 4 The Kempe Center, University of Colorado, Aurora, USA

**Keywords:** Fidelity of implementation, Evidence-based intervention, School, LikeSkills Training, Curriculum

## Abstract

As evidence-based interventions (EBIs) become more widely disseminated, fidelity of implementation (FOI) often wanes. This study explores the association between FOI and malleable variables within classrooms that could be targeted to optimize resources without compromising FOI as school-based EBIs are disseminated across real-world settings. We utilized process evaluation data from a national dissemination project of the Botvin *LifeSkills Training* (LST) middle school program, a universal prevention intervention shown to reduce substance use. The sample included 1,626 teachers in 371 schools across 14 states. Hierarchical linear models examined the relationship between observational measures of implementation factors and three domains of fidelity (e.g., adherence, student responsiveness, and quality of delivery). Findings suggest that curriculum modifications, student misbehavior, and shortage of time to implement the LST middle school program were factors most associated with lower FOI. Class size, access to program materials, and whether LST was delivered in a traditional classroom setting that is well-suited for instruction (versus in a less structured environment such as the school cafeteria) are less predictive. In scale-up of classroom-based universal interventions targeting behavioral health outcomes, our findings indicate that carefully vetting modifications, supporting classroom management strategies, and ensuring sufficient class time for implementation of highly interactive EBIs such as LST are important considerations. Since changes to EBIs are inevitable, efforts are needed to guide facilitators in making adjustments that improve program fit without compromising the essential intervention activities deemed necessary to produce desired outcomes.

## Introduction

Advances in prevention science have led to the development of evidence-based interventions (EBIs) that prevent an array of behavioral health problems (Hawkins et al., 2016). Studies suggest, however, that as EBIs become more widely disseminated, they tend to be implemented with less fidelity, or in ways that compromise their core components that account for the intervention’s efficacy (Dusenbury et al., 2005; Gottfredson & Gottfredson, 2002; Ringwalt et al., 2008), likely contributing to a “voltage drop” in outcomes (Chambers et al., 2013). In general, “implementation” refers to what an intervention consists of when it is delivered in a particular setting. Poor implementation quality can undermine EBI effectiveness (Durlak & DuPre, 2008). As such, fidelity of implementation (FOI) — or implementing an EBI as intended — has been established as essential to yielding positive participant outcomes (Chambers et al., 2013; Durlak & DuPre, 2008; Durlak, 2015). Adhering to fidelity guidelines, however, is challenging when EBIs are inevitably adapted to realities of the environment and context that exist outside of carefully controlled clinical trials (Durlak & DuPre, 2008). Specifying the “right balance” of flexibility and fidelity is a topic of much research in implementation science (Steinka-Fry et al., 2017; Stirman et al., 2019). We aim to expand this literature by examining the association between variables within the classroom level of a school system and FOI of a classroom-based EBI shown to improve a range of behavioral health outcomes among young adolescents. Understanding this relationship will inform decisions about elements of the classroom environment that can be adjusted to optimize resources without compromising FOI as school-based EBIs are disseminated across real-world settings.

This study uses data from a large-scale dissemination project of the Botvin *LifeSkills Training* (LST) Middle School program, a universal prevention program for middle school–age students generally facilitated by classroom teachers using a range of teaching techniques, including didactic instruction, discussion, demonstration, and behavior skill rehearsals to teach personal self-management skills (e.g., self-esteem, problem solving, coping), social skills (e.g., communication, building relationships), and drug resistance skills (e.g., consequences of drug use, refusal skills) (Botvin & Kantor, 2000). The EBI has demonstrated through several well-designed and well-implemented experimental studies (Steeger et al., 2021) to be effective in reducing risky behaviors and maintaining results over time. For example, findings demonstrate that LST decreases use of tobacco, alcohol, and marijuana up to 80%, with effects sustained through high school (Botvin et al., 1995, 2006). Botvin et al. (e.g., 1995) established efficacy (i.e., the extent to which an intervention does more good than harm when delivered under optimal conditions) of the LST program and Botvin et al. (2006) reported replicated efficacy results. Meanwhile, Spoth et al. (2002) established LST’s effectiveness (i.e., intervention effects when delivered in real-world conditions). State-level initiatives have shown that LST is scalable and, when implemented with fidelity, yields substantial benefits. As of 2017, LST had been adopted by over 1,200 communities serving more than one million youth with a cost–benefit ratio of $13.49 for every $1 spent (Hawkins et al., 2016; Washington State Institute for Public Policy, 2019).

Running an evidence-based program, however, is highly dynamic and often requires adjusting to changing circumstances. As Damschroder et al. (2009) explain, “The tension between the need to achieve full and consistent implementation across multiple contexts while providing the flexibility for local sites to adapt the intervention as needed is real and must be balanced, which is no small challenge” (p. 50). Implementation recommendations for LST include that lessons be implemented: (1) with program materials (i.e., teacher manuals and student guides); (2) in approximately 40–45-min class periods; (3) in a space well-suited for instruction and learning; (4) with class sizes that allow for ample discussion, skill practice, and feedback; (5) with ground rules to manage student behavior; and (6) with limited modifications to the curriculum (National Health Promotion Associates, 2017). Adjustments beyond such specified protocols are likely and efforts must therefore be made to distinguish adaptable elements, structures, and systems related to the intervention and organization into which it is being implemented (Damschroder et al., 2009; Stirman et al., 2019).

### Measuring Fidelity of Implementation (FOI)

FOI has various definitions, and many focus on the following five domains: (1) adherence, or whether program components are delivered as prescribed; (2) dosage, as in the frequency or duration of program delivery; (3) quality of delivery, such as how well the program material is implemented; (4) participant responsiveness, meaning how well the intervention is received or perceived; and/or (5) program differentiation, as in the degree of contrast between treatment and control activities (Century et al., 2010; Domitrovich et al., 2008; Dusenbury et al., 2005). Adherence and dosage are frequently reported in the literature, likely because they commonly consist of counts that are more concrete to measure (Durlak, 2015; Durlak & DuPre, 2008) and are easier to assess and interpret (Kerns et al., 2021). Evaluating fidelity of classroom-based EBIs, however, also requires consideration of how well a curriculum is implemented and received. Doing so can be problematic, as many difficult-to-measure features of instruction outside the prescribed intervention may influence outcomes and participants’ experience with the EBI, such as the extent to which “good teaching” or adaptation interacts with implementation of the EBI’s instructional model (O’Donnell, 2008; Rogers, 2003). As such, quality of delivery and student responsiveness are important, particularly in the context of classroom-based EBIs, despite these domains being difficult to measure given their complexity (Durlak, 2010; Humphrey et al., 2018). The current study follows the FOI definitions of Century et al. (2010), Domitrovich et al. (2008), and other frameworks (e.g., Dusenbury et al., 2005) for three FOI domains (i.e., adherence, quality of delivery, student responsiveness). Due to data limitations — explained below in “12” — this study does not evaluate dosage or program differentiation.

### Conceptual Frameworks for FOI of Classroom-Based EBIs

Implementing an EBI with fidelity requires considerable resources (e.g., personnel, time, external support), and for EBIs adopted in classrooms, each school’s and classroom’s context is dynamic, typically changing across and within academic years. Many theoretical frameworks exist to aid in implementation theory and evaluation, including in school settings. This study leaned on the frameworks of Domitrovich et al. (2008) to provide an overall conceptual model and Century et al. (2010) to specify malleable classroom-level variables for our analysis.

Domitrovich et al. (2008) identified two conceptually distinct components to consider regarding factors related to FOI: (1) the intervention itself and (2) the support system for the intervention. The context and environment constitute the intervention support system and, as such, must be considered within a comprehensive assessment of FOI (Domitrovich et al., 2008; Durlak, 2015). The intervention support system serves to reduce variance in high-quality implementation by providing the infrastructure necessary to implement the EBI as intended. As such, the intervention itself and the corresponding support system are interrelated. Domitrovich et al. (2008) developed a multi-level framework for considering factors of the support system influencing outcomes specific to school-based EBIs, which included the following: (1) *macro-level factors* (e.g., federal, state, district policies), (2) *school-level factors* (e.g., school characteristics, school and classroom climate), and (3) *individual-level factors*, (e.g., teacher demographics, beliefs, and attitudes). For the present study, this social-ecological framework informed both (1) the broader understanding of where and how classroom-level factors fit within the support system of a large-scale, classroom-based EBI dissemination project, and (2) which factors to include as control variables in a statistical model predicting FOI.

Malleable classroom-level factors assessed in this study were also informed by Century et al. (2010) to aid in (1) defining and measuring FOI, and (2) identifying critical factors that influence FOI. Century et al. (2010) suggest that written materials supplied by program developers and experts involved in implementation should guide dissemination of an EBI. Thus, the present study utilized guidelines established by the developer of LST (National Health Promotion Associates, 2013, 2017) to identify specific factors (e.g., material usage; time to teach the lessons; facility or space to deliver the program; ideal class size; classroom management strategies; flexible components of the curriculum that are appropriate to modify) related to FOI (i.e., whether the EBI was implemented as intended).

In the context of school-based EBIs, the Domitrovich et al. (2008) multi-level framework considers the classroom to be within the school-level sphere of influence as the school and classroom are inherently connected because classroom factors are often directly related to infrastructure and support within the school. Studies cite various factors within the school system that facilitate the success of taking to scale a classroom-based EBI, such as appropriate training, access to EBI materials, sufficient time to implement with fidelity, and modifications tailored to population fit that achieve optimal engagement without deviation from core components of the EBI (Domitrovich et al., 2008; Dusenbury et al., 2005; Gottfredson & Gottfredson, 2002; Hansen et al., 2013; Lane & Beebe-Frankenberger, 2004; Ringwalt et al., 2008). While research has explored the relationship between FOI and various macro-level factors (i.e., community norms and policies for EBIs) (Durlak & DuPre, 2008; Fagan et al., 2019; McIntosh et al., 2016), structural characteristics of a school (i.e., school size, proportion of students on free or reduced lunch and locale) (McIntosh et al., 2016; Payne & Eckert, 2010; Schaper et al., 2016), and individual characteristics of implementers (i.e., years of experience or support of the program) (Domitrovich et al., 2008; Mihalic et al., 2008; Pas et al., 2015; Wang et al., 2017), research exploring the relationship between FOI and *classroom-level factors* is limited. A few exceptions exist; for example, Mihalic et al. (2008) reported that student misbehavior was negatively associated with quality of delivery and adherence to the LST curriculum. Related to facility, the Good Behavior Game® is a classroom-based behavior management strategy shown to reduce aggressive behavior when implemented with first grade students (Dolan et al., 1993), and has been evaluated with students as old as high school (Kleinman & Saigh, 2011). Designed for traditional classroom settings, the Good Behavior Game has produced positive behavioral change even when delivered in the school cafeteria (McCurdy et al., 2009) or physical education classes (Patrick et al., 1998). Additionally, larger class sizes have been associated with poorer FOI in early childhood education (Marti et al., 2018; Zvoch, 2009). However, the association between facility or class size and FOI for a curriculum-based EBI in middle schools is unknown.

## Current Study

The current study builds on the previous literature that investigates the relationship between FOI and social-ecological factors influencing FOI of school-based EBIs, specifically those at the classroom-level and that are malleable to a facilitator of an EBI. Few data sources include detailed process evaluation from large-scale implementation of an EBI outside of a randomized trial, allowing these data to uniquely inform real-world scale-up. Classroom-level factors examined in this study are considered critical for successful EBI implementation in the literature (Century et al., 2010; Lane & Beebe-Frankenberger, 2004; Lund & Stains, 2015; Schaeffer et al., 2005) and also follow LST implementation recommendations (National Health Promotion Associates, 2017). We used multilevel modeling to empirically examine the influence of these variables on three domains of fidelity and posed the following research question: *What classroom-level factors (i.e., class size, student misbehavior, shortage of time, inadequate facility, lack of access to EBI materials, and modifications to the EBI) predict fidelity of implementation in bringing to scale a universal evidence-based behavioral intervention as measured by adherence, student responsiveness, and quality of delivery?* We hypothesized that associations between classroom-level factors and FOI would be in a negative direction; however, given the limited research on how these factors influence FOI, the question of which factors would have the strongest and most consistent associations was exploratory.

## Method

We examined process evaluation data from a national dissemination project of the Botvin *LifeSkills Training* (LST) middle school program across 14 states. A total of 127 school districts participated (representing 371 schools), and 1,626 teachers taught the LST curriculum. As part of a 3-year project, all participating schools received the same support (i.e., pre-program training, technical assistance, and implementation support), and implemented the program between academic years 2016–2017 and 2018–2019. A university institutional review board confirmed that no ethical approval was required due to the exclusive use of retrospective data that were part of routine process evaluation.

### LST Dissemination Project

The LST middle school program is a classroom-based intervention implemented in either grades 6–8 or 7–9 depending on school structure. Thirty sessions are divided into three levels, which are taught in sequence over three school years. Following the LST model, in the first year of the project, Level 1 (15 foundational sessions) was taught to 6th-grade students in the 6–8 implementation plan (or 7th in the 7–9 implementation plan), with LST taught one to five times per week. In the second year, these students received Level 2 (10 booster sessions), while an incoming cohort of 6th (or 7th)-grade students received Level 1. In the third year, 8th (or 9th)-grade students received Level 3 (5 booster sessions), while 7th (or 8th)-grade students received Level 2, and an incoming cohort of 6th (or 7th)-grade students received Level 1. Also, the curriculum has a total of nine optional violence prevention lessons (three in Level 1, two in Level 2, four in Level 3). Across the 3 years, 78% to 80% of teachers taught at least one optional lesson.

In lieu of monetary incentives, participating schools were provided with LST curriculum materials, training, process evaluation reports, and technical assistance at no cost. All teachers received a 1- or 2-day training workshop in the first year of implementation and were offered an optional 1-day booster training in the following year(s). Training was required for all LST instructors and local coordinators and encouraged for school administrators and support staff. Technical assistance was provided throughout the project, which included annual visits by consultants trained in the LST model to meet with LST teachers, classroom observers, and school administration to discuss implementation progress and problems. Additionally, phone-based and on-site technical assistance was offered as needed, generally after teachers started delivering lessons and had identified specific questions.

### Sample

Across the 14-state footprint individual schools or a collection of schools within a district responded to a Request for Proposal and applied to receive the LST program. To be eligible, schools were required to commit to implementing the program, identify a coordinator for the school district to monitor program activities, attend an LST training, and participate in the process evaluation. All applications met eligibility requirements, resulting in a sample of 127 school districts representing 371 schools. Table 1 shows descriptive statistics of the school districts. On average, a school district had three participating schools, about half were rural, and over half of students in participating schools were white. Most schools (55.3%) had three or fewer teachers for all three LST levels; 20.8% of schools had one teacher, 20.5% had two teachers, 23.9% had three to five teachers, and 26.1% had six or more teachers for all three LST levels. Though health teachers comprised the largest portion of LST facilitators, this varied by school and included social studies, science, math, computer science, and language arts teachers, as well as school counselors (18.8% of districts had a counselor teach LST). On average, LST instructors had 13.9 years of teaching experience.

**Table 1:** Descriptive statistics

	**M (SD)/%**	**Range**	**Reporter/data source**	**Brief description**
**Dependent variables, *N* = 1626**				
Adherence	73.7 (20.74)	2.50–100.00	Observer	% of LST content taught
Student responsiveness (3 items, *α* = .91)	4.25 (0.62)	1.00–5.00	Observer	Mean of items on how well students understood, participated, and responded
Quality of delivery (7 items, *α* = .96)	4.28 (0.69)	1.14–5.00	Observer	Mean of items on teacher’s knowledge, rapport, enthusiasm, classroom management, etc
**Level 1 (classroom) variables, *N* = 1626**				
Lack of materials	0.01 (0.09)	0.00–1.00	Observer	Insufficient LST materials for more than one student to participate, or teachers lacking materials
Shortage of time	0.11 (0.24)	0.00–1.00	Observer	LST lesson was truncated or unexpectedly interrupted (e.g., fire drill, assembly, early release)
Student misbehavior	0.16 (0.30)	0.00–1.00	Observer	Misbehavior so distracting that it consistently took time away from the LST lesson
Inadequate facilities	0.03 (0.15)	0.00–1.00	Observer	LST lessons occurring in any space with poor acoustics, or insufficient seating or area for activities
Modifications	0.13 (0.27)	0.00–1.00	Observer	Activity, resource, or teaching modality not prescribed by LST materials or a certified trainer (e.g., videos)
Class size	22.72 (6.21)	5.67–64.00	Observer	Teacher’s mean number of students
**Level 2 (school district-level) variables, *N* = 127**				
# of schools	2.92 (4.80)	1.00–48.00	District	Number of schools participating within the district
Rural district	55.10%	-	District	Designated locale of school district
Proportion of white students	0.59 (0.30)	0.00–0.99	District	Average percent of students who were white
Teacher support	4.11 (0.56)	2.00–5.00	Teacher	In favor of having the LST program in their school
Administrative support	4.28 (0.42)	3.00–5.00	Teacher	School administrators were supportive of LST

Level 1 classroom variables (except class size) reflect the proportion of a teachers’ observed lessons in which that classroom factor was observed

*LST Botvin* LifeSkills Training program

### Measures

Data were collected during classroom observations. Measures included a checklist created by the developer of LST (National Health Promotion Associates, 2013), and project-developed measures informed by two senior implementation scientists intimately familiar with survey design and program replication — one an internationally renowned criminologist and sociologist with over 30 years of research experience (Elliott, 2021) and the other a sociologist with expertise in implementation (Elliott & Mihalic, 2004). These measures also follow many factors outlined in the Century et al. (2010) and the Domitrovich et al. (2015) frameworks.

Classroom observers were hired for each school district to collect data for a process evaluation of the dissemination project, which consisted of completing an observation checklist and survey assessing FOI. To increase reliability, observers (who typically were not school staff) were required to attend a 1- or 2-day training workshop in the first year of implementation and were offered an optional 1-day booster training in the following year(s). Additionally, observers were required to participate in a 60–90-min training session dedicated to the procedures and protocols of the observer role. A total of 276 observers completed both training components and were instructed to observe each Level 1 or multi-level teacher four times, each Level 2 teacher three times, and each Level 3 teacher two times. Though scheduling conflicts, teacher turnover, and other challenges sometimes affected the number of recommended observations, over the 3-year project, teachers were observed on average 3.8 times (*SD* = 3.3), depending on which level(s) or grades of LST they taught each year.

#### Dependent Variables

This study measured three domains of fidelity per various frameworks (e.g., Century et al., 2010; Domitrovich et al., 2008; Dusenbury et al., 2005). Average scores were constructed for each teacher across the 3-year project for each dependent variable. (1) *Adherence* was defined as the percentage of content taught in observed lessons, and was measured through a checklist created by the developer of LST (National Health Promotion Associates, 2013). Observers indicated the percentage of activities and lesson points in the teacher’s manual covered by the instructor. This checklist has been used in numerous LST evaluations and replication projects and has demonstrated inter-rater reliability above 0.80 (Botvin et al., 1995; Mihalic et al., 2008). During annual site visits, consultants conducted joint classroom observations with the local observers. Across the 3 years, a total of 381 co-observations were conducted. Because these data were originally collected for the purpose of a process evaluation, data were collected and stored to assess inter-rater agreement only (i.e., the number of agreement scores divided by the total number of scores) and not kappa (which would take into account agreement by chance) (McHugh, 2012). We found that, on average, observers and consultants agreed on 90.9% of the curriculum points taught during the observed session. (2) *Quality of delivery* was measured through seven project-developed items assessing the instructor’s delivery of lessons including: teachers’ knowledge of the program, enthusiasm, poise and confidence, rapport and communication, classroom management, ability to address questions and concerns, and overall quality of the lesson. Response options were on a Likert scale (1 = Poor to 5 = Excellent) and had strong internal reliability for the sample (*α* = .96). The items were averaged to create a quality of delivery score. (3) *Student responsiveness* was assessed through three project-developed items asking how well students understood, participated in, and responded to the lesson. Response options were on a Likert scale (1 = Poor to 5 = Excellent), and demonstrated high internal reliability (*α* = .91). The three items were averaged to create a student responsiveness score. Items assessing quality of delivery and student responsiveness were similar to those in previous studies (Humphrey et al., 2018; Rohrbach et al., 2010; Vroom et al., 2020).

#### Classroom-Level Independent Variables

In addition to measuring direct indicators of FOI, the project-developed survey completed during classroom observations recorded factors critical to FOI per LST implementation recommendations (National Health Promotion Associates, 2017) and implementation science experts described above. On top of assessing the setting in which LST is implemented, to compare to ideal conditions as defined by the developer, these classroom-level factors fall within the broader school level and specifically the classroom level of the Domitrovich et al. (2008) multi-level framework. The framework outlined in Century et al. (2010) further informed the specifics of these classroom-level factors. Accordingly, for each observation, observers recorded the number of students in attendance and indicated (yes or no) if the below five factors occurred during the lesson.

*Lack of materials* was defined as issues such as more than one student not having a copy of the student guide, insufficient materials for most students to participate in an activity, or teachers lacking necessary instructional materials (i.e., teacher’s manual or student guide). *Inadequate facility* included lessons occurring in any facility or room with poor acoustics, insufficient desk or table space, or insufficient space for skill practice activities (e.g., crowded classroom, weight room, band room, gym, cafeteria, outdoors). *Shortage of time* reflected when the LST lesson was unexpectedly interrupted due to a school drill (e.g., fire, active harmer, tornado), or shortened either due to a schedule conflict (e.g., delayed start, early release, assembly) or because class time was used to cover material not related to LST. Observers were trained to indicate *student behavior* as a problem only when misbehavior was so distracting that it consistently took time away from the lesson (i.e., other students were distracted, and therefore the teacher had to repeatedly interrupt the lesson to redirect student behavior). *Modifications* were defined as any activity or teaching modality observed that was not prescribed by the LST teacher’s manual or student guide or suggested by a certified LST trainer. Examples included adding videos, guest speakers, and covering other content outside of the LST curriculum (e.g., “drunk goggles,” displays of black lungs). For each teacher, five scores were calculated to represent the proportion of observed lessons across 3 years in which a lesson was modified, disrupted by student misbehavior, taught in an inadequate facility, lacked program materials, or was interrupted causing a shortage of time. The final classroom-level variable, *class size*, was calculated for each teacher as their mean number of students across the three project years. There was no missingness in observer-reported data.

#### School District-Level Control Variables

Table 1 displays school district-level control variables that were theoretically pre-determined based on our literature review, conceptual frameworks (Century et al., 2010; Domitrovich et al., 2008), and data available. School districts provided information on characteristics of their schools, specifically the percentage of students identifying as white or as Black, Indigenous, or Persons of Color (BIPOC), whether the district was mostly rural (0) or urban/suburban (1), and the number of schools participating in their district. Using the National Center for Education Statistics’ designations, locale was later confirmed for general consistency across school districts, as well as for school districts that selected multiple locale descriptions or left the item blank. Specifically, the number of schools implementing in the district reflected macro-level factors, while locale and race reflected school-level factors per Domitrovich et al. (2008). Also, teachers were invited to complete an end-of-year survey, which had a 74% response rate across the 3 years. Teacher and administrator support for LST was assessed by asking teachers how much they agreed with the following statements*: I am in favor of having the LST program in my school; School administrators were supportive of the LST program* (response options: 1 = Strongly disagree, 5 = Strongly agree). Average scores for teacher and administrator support were aggregated by district and reflected individual or implementer characteristics per Domitrovich et al. (2008). All districts were represented, and there were no missing data. District-level means for classroom-level predictors also were included in models to control for potential contextual effects (i.e., incremental effects of district characteristics after controlling for classroom characteristics) and for proper interpretation of the level 1 predictors (Hoffman & Walters, 2022).

## Analysis

Two-level hierarchical linear models were conducted in HLM 7.01 to account for the nesting of teachers within school districts. Three separate models (with random intercepts and fixed slopes) were run for each outcome (i.e., adherence, student responsiveness, quality of delivery), with classroom variables at level 1 and district variables at level 2. All models included the same set of theoretically pre-determined predictors, and continuous predictors were grand mean centered. District, rather than school, was the level 2 clustering variable for several reasons. First, there was an insufficient number of teachers within schools to account for clustering between schools (over one-half of schools had one to three teachers, or one teacher per level/classroom of LST taught). Secondly, schools within districts shared similar characteristics and had the same administration and LST coordinator; thus, schools within each district experienced similar conditions for implementing LST.

Intraclass correlations (ICCs) were estimated for each outcome variable using an empty model (i.e., random intercept only and no predictors). The ICCs showed 17.5% of variability in adherence, 14.1% in quality, and 13.3% in responsiveness, which were due to differences between school districts. ICCs can also be interpreted as the correlation among observations within the cluster; in this case, the correlation between teachers within a school district. We reported pseudo-*R*^2^ (Snijders & Bosker, 1999), deviance statistics, and robust standard errors. All variables were standardized prior to analyses in HLM; this produces standardized coefficients that provide information about the magnitude of the effect and are comparable across models and studies with similar populations (Lorah, 2018). Thus, coefficients can be interpreted as the predicted change in standard deviations on the outcome, resulting from a one standard deviation increase in a predictor. The mixed model equation is shown in (1):

1 $$\begin{aligned}Outcome_{j}\:&=\:\gamma_{00}\:+\:\gamma_{01}\;\ast\;level\;2:\;lack\;materials_{j}\:\\&+\:\gamma_{02}\;\ast\;level\;2:\;shortage\;time_{j}\:\\&+\:\gamma_{03}\;\ast\;level\;2:\;misbehavior_{j}\:\\&+\:\gamma_{04}\;\ast\;level\;2:\;facility_{j}\:\\&+\:\gamma_{05}\;\ast\;level\;2:\;modification\:\\&+\:\gamma_{06}\;\ast level\;2:\;class\;size_{j}\:\\&+\:\gamma_{07}\;\ast\;level\;2:\;\%\;white_{j}\:\\&+\:\gamma_{08}\;\ast\;level\;2:\;rural_{j}\:\\&+\:\gamma_{09}\;\ast level\;2:\;schools_{j}\:\\&+\:\gamma_{010}\;\ast\;level\;1:\;admin\;support_{j}\:\\&+\:\gamma_{011}\;\ast\;level\;1\;support_{j}\:\\&+\:\gamma_{10}\;\ast level\;1:\;lack\;materials_{ij}\:\\&+\:\gamma_{20}\ast\;level\;1:\;shortage\;of\;time_{ij}\:\\&+\:\gamma_{30}\;\ast\;level\;1:\;misbehavior_{ij}\:\\&+\:\gamma_{40}\;\ast\;level\;1:\;facility_{ij}\:\\&+\:\gamma_{50}\;\ast\;level\;1:\;modification_{ij}\:\\&+\:\gamma_{60}\;\ast\;level\;1:\;class\;size_{ij}\:+\:u_{0j}\:+\:r_{ij}\end{aligned}$$

## Results

Table 1 displays descriptive statistics for all dependent and independent variables. As shown in Table 2, for each model, deviance statistics decreased from the empty to the full models. Proportion of variance (i.e., pseudo-*R*^2^) explained in the dependent variables ranged from 4.0% for adherence to 18.1% for student responsiveness. Three of the six classroom-level factors were inversely associated with adherence: shortage of time (*B* = −0.10, *p* < .001), student misbehavior (*B* = −0.10, *p* < .001), and modification to the lesson (*B* = −0.14, *p* < .001) were associated with fewer curriculum points covered. Meanwhile, three classroom factors were inversely associated with student responsiveness: increased misbehavior (*B* = −0.39, *p* < .001), modifications (*B* = −0.09, *p* < .001), and shortage of time (*B* = −0.05, *p* = .026) were associated with lower student responsiveness. Finally, three classroom factors were inversely associated with quality of delivery: increased student misbehavior (*B* = −9.46, *p* < .001), modifications (*B* = −0.10, *p* < .001), and lack of materials (*B* = −0.07, *p* = .029) were associated with lower quality.

**Table 2:** Hierarchical linear models of associations between classroom-level implementation factors and fidelity of implementation

**Variable**	**Adherence**			**Responsiveness**			**Quality**			
	***B***	**SE**	***p*-value**	***B***	**SE**	***p*-value**	***B***		**SE**	***p*-value**
Level 2 (School district) fixed effects										
Intercept	0.17	0.07	0.016	0.08	0.06	0.192	0.15*		0.06	0.011
% materials	−0.10	0.05	0.072	−0.03	0.03	0.241	−0.003		0.03	0.909
% shortage time	0.03	0.06	0.625	0.02	0.04	0.585	0.05		0.05	0.311
% misbehavior	−0.02	0.05	0.718	0.01	0.05	0.901	−0.01		0.05	0.797
% inadeq. facility	−0.03	0.05	0.528	−0.15**	0.05	0.005	−0.12*		0.05	0.020
% modifications	0.08	0.04	0.066	0.01	0.03	0.764	0.04		0.03	0.284
District class size	-0.004	0.07	0.951	0.04	0.05	0.415	0.03		0.05	0.581
% White	0.08	0.05	0.152	0.20***	0.04	< 0.001	0.16***		0.04	< 0.001
Rural	−0.09	0.11	0.392	0.10	0.09	0.245	−0.04		0.10	0.711
# of schools	−0.04*	0.02	0.010	−0.02*	0.01	0.029	−0.01		0.01	0.279
Admin. support	−0.09	0.06	0.129	−0.01	0.05	0.790	−0.03		0.05	0.506
Teacher support	0.05	0.07	0.423	0.12*	0.05	0.012	0.12*		0.05	0.016
Level 1 (Classroom) fixed effects										
% lack materials	−0.02	0.03	0.572	−0.04	0.03	0.150	−0.07*		0.03	0.029
% shortage time	−0.10***	0.02	< 0.001	−0.05*	0.02	0.026	−0.03		0.02	0.205
% misbehavior	−0.10***	0.03	< 0.001	−0.39***	0.03	< 0.001	−0.38***		0.04	< 0.001
% inadeq. facility	0.0001	0.03	0.996	−0.02	0.04	0.698	0.001		0.04	0.981
% modifications	−0.14***	0.03	< 0.001	−0.09***	0.02	< 0.001	−0.10***		0.02	< 0.001
Class size	0.04	0.05	0.422	0.04	0.02	0.091	0.04		0.02	0.136
Random effects										
Variance component										
Intercept	0.13			0.04			0.06			
Level 1	0.82			0.72			0.72			
Goodness of fit										
Deviance	4431.60			4165.79			4193.39			
*Δ χ*^2^	36.68^**^			324.19^***^			289.50^***^			
Proportion of variance explained										
Pseudo-*R*^2^	.040			0.181			0.168			

The dependent variables were standardized for analyses. Goodness of fit tests showed statistically significant decreases in deviance statistics from unconditional to the full models; *Δ df* for all *Δ χ*^2^ difference tests were 17

^*^p < .05; ** *p* < .01; ***p < .001

## Discussion

As evidence-based interventions (EBIs) become more widely disseminated, fidelity of implementation (FOI) often wanes. To better understand this phenomenon and how to effectively scale-up EBIs in ways that maintain FOI, this study explores the association between FOI and malleable variables within the classroom level of a school system, while controlling for other factors that may be associated with implementation more generally. We expand upon the literature investigating socio-ecological factors, and specifically classroom-level factors, influencing FOI (Domitrovich et al., 2008) by utilizing process evaluation data from a national dissemination project of the Botvin LifeSkills Training (LST) middle school program, a universal prevention intervention shown to reduce substance use and violence (Botvin et al., 1995, 2006). Our research examined classroom factors that are considered critical for effective implementation according to FOI frameworks (Century et al., 2010) and LST implementation recommendations (National Health Promotion Associates, 2017). While these frameworks and guidelines articulate constructs of relevance, they do not indicate their relative importance. Research documents nearly 30 socio-ecological factors that influence the various FOI domains across a range of EBIs (Damschroder et al., 2009; Domitrovich et al., 2008; Durlak, 2015). In the present study, we examined six classroom-level factors and found that three were consistently and strongly associated with FOI, as measured by adherence, student responsiveness, and quality of delivery. Specifically, results indicate a strong inverse relationship between FOI and modification to the curriculum, student misbehavior, and shortage of time.

The process of implementing EBIs is dynamic, and thus thoughtful and deliberate alteration to the delivery of an EBI or to the curriculum to improve its fit in a given context (i.e., adaption) can lead to improved engagement, acceptability, and outcomes (Damschroder et al., 2009; Stirman et al., 2019). However, modification to a curriculum that removes key elements of an intervention may be less effective (Stirman et al., 2019). Using a traffic light as an analogy, Balis et al. (2021) assign a color for making changes to an EBI: tailoring language or pictures (green/low risk), adding/substituting activities or session sequence (yellow/medium risk), or deleting lessons and decreasing session length (red/high risk). In the present study, we defined “modification” as “any activities, or content covered, that were not provided in the LST teacher’s manual or student guide or suggested by a certified LST trainer.” These would largely be considered “yellow light changes,” which may explain why more modification related to statistically significant decreases in adherence, quality of delivery, and student responsiveness.

Green light changes, meanwhile, are often necessary to improve program fit, and could increase cultural appropriateness (e.g., tailoring language and pictures; Balis et al., 2021). For example, Promoting First Relationships® is a mental health training program for workers in home-visiting and early care and education settings designed to promote healthy relationships between caregivers and young children from birth to 3 years of age (Oxford et al., 2016). Booth-LaForce et al. (2020) found an adapted version of this EBI to be effective when piloted with American Indian families living on a rural reservation. In collaboration with the tribal community, the program was adapted to increase cultural relevance, which included a unique name and logo by a Native artist, longer home visits to include more time for conversation, and a small gift for the child at research visits. Since modifications beyond controlled trials are inevitable (Dusenbury et al., 2005; Ringwalt et al., 2008), and EBIs can be appropriately adapted to improve fit, teachers should be guided through training and supervision to ensure adaptations do not modify core components. Meanwhile, EBI developers should evaluate which curriculum elements are “essential” and which ones can be modified without jeopardizing outcomes.

Student misbehavior was also a strong predictor of the three FOI domains. Specifically, it was significantly associated with lower adherence, student responsiveness, and quality of delivery, indicating the importance of effective classroom management strategies to support student learning within highly interactive EBIs like LST. As such, administrators may benefit from carefully selecting instructors who are skilled in facilitation and classroom management. Schools also may consider utilizing a co-facilitation approach to allow for a prompt response to problematic behavior by one staff member, while the other maintains student engagement and continuity of instruction. To minimize misbehavior, teachers can reinforce positive behaviors, plan for how to handle transitions, and establish clear routines and expectations with their students to promote connection, consistency, and compassion. Funders of EBIs and schools adopting an EBI may benefit from budgeting in technical assistance to offer strategies to help mitigate challenging student behavior. Further, shortage of time was associated with lower adherence and student responsiveness, underscoring the importance of ensuring that teachers have adequate time, without interruptions, to teach curriculum-based EBIs.

Another consistent finding was that inadequate facilities and class size were unrelated to FOI. Additionally, lack of program materials was inversely associated only with quality of delivery, and the strength of this association was weaker. Guidance from the developer of LST recommends the curriculum be delivered in a traditional classroom, with up to 25 students, in roughly 40–45-min class periods, and by means of a teacher’s manual and consumable student guides (National Health Promotion Associates, 2017). Nevertheless, when navigating multiple challenges in the classroom, the present study suggests that facility, class size, and having all materials are not as critical to FOI of a classroom-based EBI targeting non-academic outcomes (e.g., substance use) as student behavior, modification, and time to teach the curriculum.

### Limitations

Results of this study provide insight into under-reported malleable variables within the classroom level of a school system that potentially can be adjusted without negatively impacting FOI of a classroom-based EBI. There are, however, some important limitations that overall call for more studies within this scope. First, this is correlational research; as such, findings indicate statistical associations and do not establish causality. Second, while observer-reported measures are advantageous and considered more objective compared to teacher self-reports or data collected at the end of implementation (Durlak, 2015), inconsistencies may exist in observational assessments of FOI — particularly given the large number of observers. While we used inter-rater agreement to assess interrater reliability for one of three outcomes (i.e., adherence), it does not account for the random chance of guessing and thus may overestimate true agreement. The kappa statistic, which accounts for random chance of agreement (McHugh, 2012), was unable to be calculated (see “9” for details). Further, lacking measures of interrater reliability for other variables is a limitation because the degree of error from observers interpreting variables differently is unknown (McHugh, 2012). These limitations were mitigated by ensuring all observers were trained in assessing FOI of the LST model, as well as by project consultants who promptly reviewed submitted observation forms, followed up with observers to receive clarifying information, and provided feedback and guidance to promote consistency, reduce drift, and minimize guessing among raters. Third, the classroom-level factors (i.e., level 1 predictors) were assessed using single-item measures that are unlikely to fully capture the complexity of the underlying constructs. For example, *lack of materials* could relate to teacher instructional materials, student guides, or both. Finally, though this study represented more naturalistic conditions than data from highly controlled trials, these findings are within the context of schools and teachers receiving substantial support to implement the EBI. Pre-program training was provided at no cost and technical assistance was given as requested throughout the project.

Several limitations also relate to the measurement of FOI domains included in our study, namely the exclusion of dosage and program differentiation. In initial analyses, we included dosage as the average length of observed lessons in minutes. Lesson length has been used to assess dosage in the FOI literature (Domitrovich et al., 2015) and is how the developer of LST assessed dosage (Botvin et al., 2018). However, in this process evaluation, time spent on LST was an imperfect measure to capture the amount of the curriculum that students received, as the length of a lesson was constrained by the length of the class period, which was determined by the school. It was likely that teachers carried lessons over to the next class, or utilized more classes, if they were limited by a short class period, which would not be reflected in the length of the *observed* lesson. Our model using “time spent on observed LST lessons” indicated that this measure for dosage was incomplete according to poor model fit, small proportion of variance explained, and few statistically significant predictors. Additionally, we could not evaluate program differentiation as data originated from a process evaluation that did not necessitate a control group. As we did not have student outcome data, this study can only show associations between FOI domains and classroom- and district-level variables; we were unable to assess if FOI, or the classroom factors measured, had any association with student outcomes.

### Future Research and Conclusions

Adjustments are inevitable when interventions are implemented in natural settings (Chambers & Norton, 2016), yet it is challenging to both adhere to fidelity guidelines and adapt to realities of the environment. The appropriate balance of flexibility and fidelity is a topic of much research in implementation science (Steinka-Fry et al., 2017; Stirman et al., 2019). Integrating clear guidance and examples of culturally relevant curriculum adaptations into EBI materials, while indicating which aspects of implementation are most essential, may facilitate adjustments that enable strong implementation in less than ideal circumstances as well as maintain (or improve) both participant engagement and student outcomes without threatening causal mechanisms accounting for an EBI’s efficacy (Dusenbury et al., 2005; Gottfredson & Gottfredson, 2002; Ringwalt et al., 2008). Stirman et al. (2019) developed a framework for reporting adaptations and modifications to EBIs, including consideration of when and how modifications occurred, whether it was planned, and goals of modifications. To support research on modifications to EBIs, future research should record modifications (e.g., using frameworks such as Stirman et al., 2019 or Balis et al., 2021) and empirically examine associations with FOI. Documenting these changes may illuminate the “black box” of adjustments that lead to higher engagement (i.e., as “green light changes”) without jeopardizing outcomes.

Finally, this study provides clear takeaways regarding malleable factors that influence FOI. Given the many challenges in disseminating EBIs, our results offer insight into which considerations may be most important to target. Ultimately, in scale-up of classroom-based universal interventions focused on improving behavioral health outcomes, carefully vetting modifications to the curriculum, supporting classroom management, and dedicating sufficient time to the EBI are important considerations for FOI.

## Data Availability

Available from authors upon request.
